# Cognitive-motor telerehabilitation in multiple sclerosis (CoMoTeMS): study protocol for a randomised controlled trial

**DOI:** 10.1186/s13063-022-06697-9

**Published:** 2022-09-14

**Authors:** Delphine Van Laethem, Frederik Van de Steen, Daphne Kos, Maarten Naeyaert, Peter Van Schuerbeek, Miguel D’Haeseleer, Marie B. D’Hooghe, Jeroen Van Schependom, Guy Nagels

**Affiliations:** 1 AIMS Lab, Center For Neurosciences, UZ Brussel, Vrije Universiteit Brussel, Pleinlaan 2, 1050 Brussel, Belgium; 2 Department of Physical and Rehabilitation Medicine, UZ Brussel, Brussel, Belgium; 3 The Wellcome Trust Centre for Neuroimaging, University College London, London, UK; 4 Department of Rehabilitation, National Multiple Sclerosis Center, Melsbroek, Belgium; 5 Department of Rehabilitation Sciences, KU Leuven, Leuven, Belgium; 6 Department of Radiology, UZ Brussel, Brussel, Belgium; 7 Neurology Department, National Multiple Sclerosis Center, Melsbroek, Belgium; 8 Neurology Department, UZ Brussel, Brussel, Belgium; 9 Center for Neurosciences, Vrije Universiteit Brussel, Brussel, Belgium; 10 Department of Electronics and Informatics (ETRO), Vrije Universiteit Brussel, Brussel, Belgium; 11 St Edmund Hall, University of Oxford, Oxford, UK

**Keywords:** Cognition, Multiple sclerosis, Rehabilitation, Cognitive rehabilitation, Cognitive-motor rehabilitation, Telerehabilitation, Randomised controlled trial

## Abstract

**Background:**

The management of cognitive impairment is an important goal in the treatment of multiple sclerosis (MS). While cognitive rehabilitation has been proven to be effective in improving cognitive performance in MS, research in the elderly indicates a higher effectiveness of combined cognitive-motor rehabilitation. Here, we present the protocol of a randomised controlled clinical trial to assess whether a combined cognitive-motor telerehabilitation programme is more effective in improving working memory than only cognitive or motor training.

**Methods/design:**

The CoMoTeMS-trial is a two-centre, randomised, controlled and blinded clinical trial. A total of 90 patients with MS will receive 12 weeks of either a combined cognitive-motor telerehabilitation programme or only cognitive or motor training. The primary outcome is a change in the digit span backwards. Secondary outcomes are other cognitive changes (Brief International Cognitive Assessment for Multiple Sclerosis and Backward Corsi), Expanded Disability Status Scale (EDSS), 6-Min Walk Test, 25-Foot Walk Test, 9-Hole Peg Test, anxiety and depression, fatigue, quality of life, cognitive and physical activity level, electroencephalography and magnetic resonance imaging of the brain.

**Discussion:**

We hypothesise that the improvement in digit span backwards after 12 weeks of treatment will be significantly higher in the group treated with the combined cognitive-motor telerehabilitation programme, compared to the groups receiving only cognitive and only motor training.

**Trial registration:**

ClinicalTrials.gov NCT05355389. Registered on 2 May 2022.

**Supplementary Information:**

The online version contains supplementary material available at 10.1186/s13063-022-06697-9.

## Introduction

Multiple sclerosis (MS) is the most common inflammatory and neurodegenerative disease in young adults, affecting more than two million people worldwide [1], and between 34 and 65% of the persons with multiple sclerosis (PwMS) suffer from cognitive impairment (CI) [2]. CI in MS has a substantial, often detrimental effect on numerous aspects of daily life [3–5]. Its management is an important gap in the treatment of MS [6]. Cognitive rehabilitation is the most promising treatment strategy [6], with recent meta-analyses finding conclusive evidence that both cognitive rehabilitation interventions in general and memory rehabilitation improve objective cognitive performance [7–9]. However, many challenges remain that hamper implementation into daily practice; studies with larger sample sizes, inclusion of patients with progressive disease, assessment of the effects on daily life and long-term follow-up data are still lacking [6].

The link between CI and physical performance is of particular interest. Studies have found numerous associations between aspects of cognition and measures of physical performance in PwMS [10–15]. These associations impelled recent interest in the possibility of enhancing cognition by training and improving physical performance and vice versa [6, 16]. There is preliminary evidence of improved processing speed, learning, memory and executive functions in PwMS after exercise training [6]. Literature on combined cognitive-motor interventions is scarce. A few studies have also found promising results in walking performance under dual-task conditions after dual-task training [17–19]. Two studies on combined cognitive-motor training as two separate interventions found that adding a cognitive treatment to a motor treatment improved cognitive, emotional [20, 21] and motor aspects [20] in PwMS. Ageing studies on combined cognitive-motor rehabilitation are more prevalent, with limited evidence for its effectiveness in improving cognition and functional status, both with and without the presence of CI [22–24]. However, more well-designed studies with active control groups and larger sample sizes, as well as data on the appropriate training characteristics and long-term effects, are needed before definite conclusions can be drawn.

Furthermore, there is emerging evidence that telemedicine and telerehabilitation in MS are beneficial, cost-effective and satisfactory for both patients and healthcare professionals [25], as well as low-level evidence for improvement of functional activities, fatigue and quality of life after telerehabilitation in PwMS [26]. In the context of the COVID-19 pandemic, telemedicine has gained momentum and is increasingly applied in daily practice in the follow-up of MS and other conditions [27–29] and could be of particular interest in the assessment and treatment of cognitive impairment [28].

We set up a randomised controlled clinical trial to assess whether a combined cognitive-motor telerehabilitation programme improves working memory compared to cognitive or motor telerehabilitation only in PwMS in patients with impaired working memory. Furthermore, we will assess the effect on walking performance and identify mechanisms of improvement and predictors of treatment response.

## Methods

This protocol was constructed using the SPIRIT reporting guidelines ([30], see attachment). Based on these guidelines, the following diagram was constructed:

**Table:** 

	Study period							
	Enrolment	Allocation	Intervention		Follow-up			Close-out
**Timepoint**	***-T***_***1***_	**T0**	***T***_***1***_ ***= week 1***	***T***_***2***_ ***= week 12***	***T***_***3***_ ***= week 13***	***T4 = week 24***	***T***_***5***_ ***= week 64***	***T***_***6***_ ***= week 65***
**Enrolment**								
**Eligibility screen**	X							
**Informed consent**	X							
***Allocation***		X						
**Intervention preparation**		X						
**Interventions**								
***Cognitive-motor training***								
***Cognitive training***								
***Motor training***								
**Assessments**								
***Patient characteristics***	X	X						
***Neuropsychological /physical tests***	X	X			X	X	X	
***EEG***		X			X			
***MRI***		X			X	X		

### Trial design

CoMoTeMS (Cognitive-Motor Telerehabilitation in Multiple Sclerosis) is a two-centre, randomised, controlled, parallel-group and blinded clinical trial, consisting of one intervention group and two active control groups. Recruitment will start in July 2022 in the Universitair Ziekenhuis Brussel (academic hospital) and the National Multiple Sclerosis Center Melsbroek (specialist hospital). In total, 90 patients with clinically definite multiple sclerosis (based on the revised McDonald criteria 2017 [31]) will be randomised to receive either combined cognitive-motor training, cognitive training or motor training, stratified by age, sex, education and baseline activity level and cognition. This study was approved by the ethics committees of the Universitair Ziekenhuis Brussel (B.U.N. 1432022000107) and the National Multiple Sclerosis Center Melsbroek and is registered at ClinicalTrials.gov (NCT05355389, 2 May 2022). The study will be conducted in accordance with the Declaration of Helsinki, the guidelines of the International Conference on Harmonization of Good Clinical Practice (ICH-GCP) and the applicable Belgian legislation. All participants are required to give written informed consent before inclusion.

### Participants

Dutch- and/or French-speaking patients with MS will be recruited at the Universitair Ziekenhuis Brussel and the National Multiple Sclerosis Center Melsbroek, as well as through an advertisement printed in the Flanders MS society magazine and published on social media. The inclusion criteria for participation are clinically definite multiple sclerosis (revised McDonald criteria 2017, any disease course), Expanded Disability Status Scale (EDSS) [32] below 6.0, digit span backwards *z*-score between [−3 and −0.5] standard deviations below the median of the normative values [33, 34], age between 18 and 65 and ability to safely perform the motor rehabilitation programme in the home situation (assessed by a rehabilitation physician and/or occupational or physiotherapist). Exclusion criteria are participation to a cognitive rehabilitation programme within 6 months before inclusion, inpatient multidisciplinary rehabilitation programme within 3 months before inclusion or planned inpatient rehabilitation programme during trial, start of or switch in immunomodulatory treatment within 3 months prior to inclusion, less than 1 month post-exacerbation, major psychiatric or medical disorder that could influence cognitive functions, combined vision with optimal correction below 0.6 on Snellen Visual Acuity Test and the patient being unable or unwilling to undergo electroencephalography (EEG) or magnetic resonance imaging (MRI). Patients following physiotherapy at home or an outpatient multidisciplinary rehabilitation programme will not be excluded, but will be asked to postpone significant changes in intensity and content until after the trial. Furthermore, we will ask the patients to postpone inpatient multidisciplinary rehabilitation programmes from the start of the training until after the last MRI at 3 months post-training, i.e. a total period of 6 months.

### Intake and intervention

**Fig. 1:**
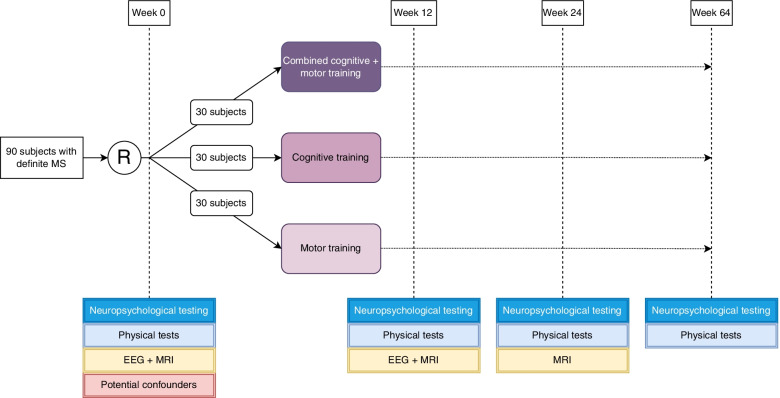
Trial flowchart

Eligibility for participation is determined at a screening visit after written consent, at which point a digit span backwards and an assessment of vision using the Snellen Visual Acuity test are performed. From patients who are eligible for participation, data on age, sex, disease type, course and duration, EDSS score, time since last relapse, medication, level of education, living situation and additional physiotherapy/rehabilitation will be collected. Furthermore, they will be randomised 1:1 to receive either combined cognitive-motor training, cognitive training or motor training, stratified by age, sex, education, baseline activity level and cognition (through minimisation of the intercentroid distances in a five-dimensional space). Each group will receive 90 min of training per week for 12 weeks. This duration and frequency are based on a systematic review on cognitive-motor rehabilitation in the elderly, which recommends a training scheme of 1 to 3 h weekly for 12 to 16 weeks [23]. Furthermore, clinical and neuroimaging assessments will be planned before and after the training programme, at 24 weeks and at 64 weeks (see Fig. 1). Neuropsychological and physical testing and EEG will take place 1 week before the start and 1 week after completion of the training. MRI measurements will be carried out in a time window of 2 weeks before and after the training.

Patients in the cognitive-motor training group will carry out both the cognitive and the motor training programme, while patients in the cognitive training group and the motor training group will respectively carry out only the cognitive training programme and the motor training programme (see Fig. 1). Before the start of the training, a start-up teleconsultation with an MS nurse will be scheduled, where patients will receive clear instructions on how to carry out the training programme(s).

For the cognitive treatment intervention, we will use the widely used cognitive training programme RehaCom® (Hasomed GmbH, Magdeburg, Germany). This is a computer-aided programme with more than 30 modules focusing on different domains of cognition. RehaCom has shown improvements in verbal learning, visuospatial memory, information processing speed, attention, executive functions, depression, fatigue and quality of life in PwMS [35–38]. Patients will train on their home computer without direct therapist supervision, using three RehaCom modules (Working Memory, Topological Memory and Shopping) that are focused on improving working memory. Both patients in the combined intervention and the cognitive intervention group will be doing a 45-min computer session respectively 1 and 2 days per week for a total of 12 weeks. During one 45-min training session, patients will complete 15 min of exercises of each of the three modules. The level of difficulty of the exercises is automatically adapted to the patient’s performance.

**Fig. 2:**
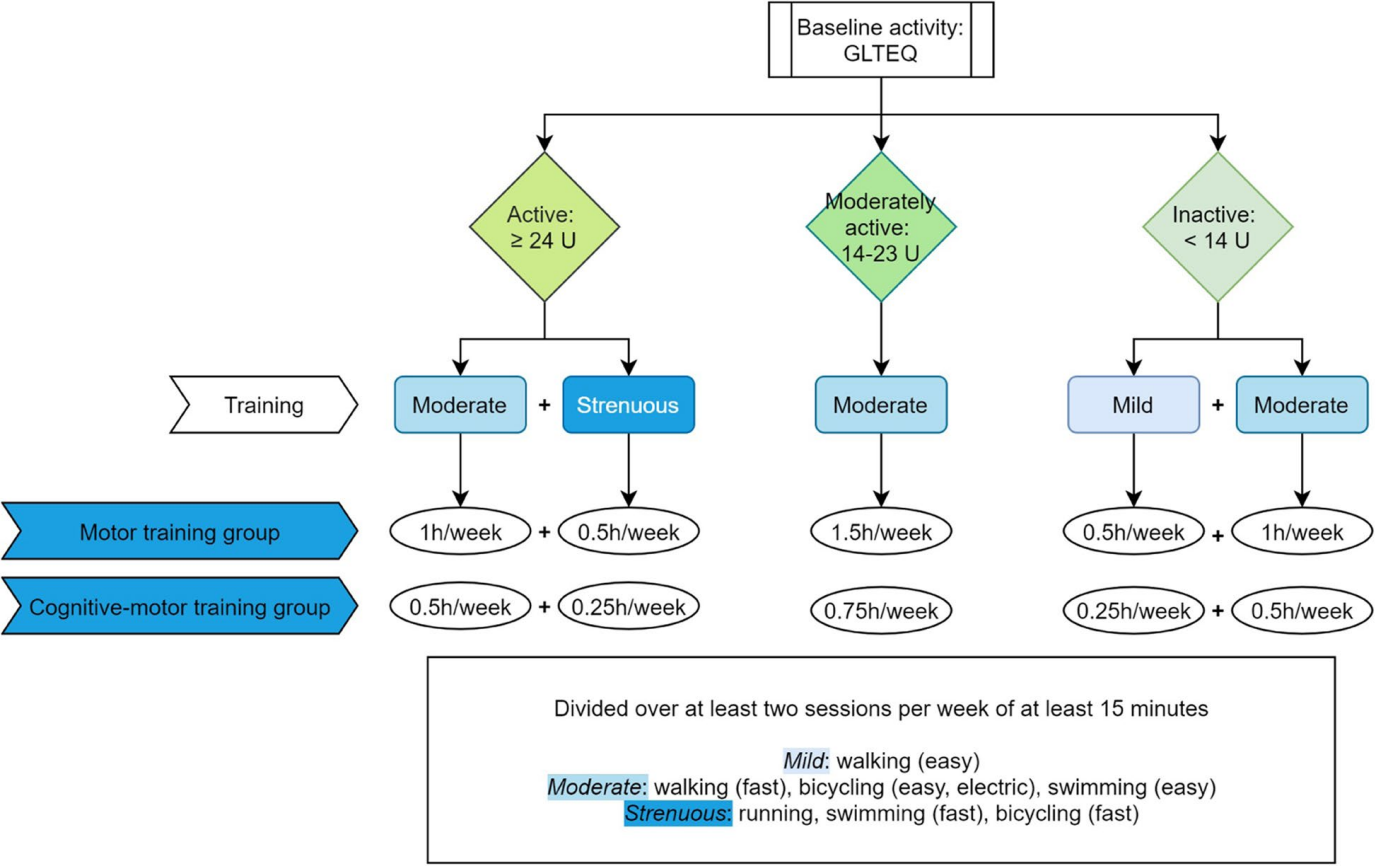
Motor training algorithm

For the motor training, we will use a patient-tailored training programme based on the patient’s baseline activity level. At inclusion, all patients will complete the Godin Leisure-Time Exercise Questionnaire (GLTEQ) to assess their level of activity and be divided into three groups (see Fig. 2) [39]. Based on their baseline physical activity level, patients can choose from a number of aerobic activities of either mild, moderate or strenuous intensity, with a total training time of 90 min per week for the motor training group and 45 min for the combined cognitive-motor training group, divided over at least two training sessions per week of at least 15 min per session (see Fig. 2). The training will be carried out individually at home, without therapist supervision. Patients will use a heart rate sensor to maintain the correct training intensity, with 20–39%, 40–59% and 60–84% of their heart rate reserve (target heart rate = heart rate reserve*X% intensity + resting heart rate; heart rate reserve = *maximum heart rate − resting heart rate*; *maximum heart rate = 220 − age* [40]) respectively corresponding to mild, moderate and strenuous training [41–43]. Heart rate will be monitored using a sport watch (Polar Unite, Polar Electro, Kempele, Finland), provided by the research team and equipped with an accelerometer and a heart rate sensor. Furthermore, patients will be asked to rate the training intensity with the Rating of Perceived Exertion scale (RPE [44]). A score of 10–11, 12–13 and 14–16 corresponds to mild, moderate and strenuous training respectively [41, 42]. Both baseline activities and training activities in the context of the study will be logged using the sports watch. In order to ensure that activity levels remain the same in the group receiving only cognitive training, the activity levels of these patients will be monitored as well. Adaptations to training intensity in response to issues reported by patients will be decided on a case-by-case basis.

Participants will have a 20-min teleconsultation once every 2 weeks with a trained MS nurse. The teleconsultations will take place using Microsoft Teams® (Microsoft Corporation, Redmond, WA, USA) and the patient can use any device equipped with a webcam and microphone. The consultations consist of a structured interview dealing with motivation of the patient to continue the training, perceived impact of cognitive and physical difficulties on the patient’s life in the past weeks, effort applied to the training in the previous weeks, technical difficulties experienced, targets for next weeks’ training, and additional questions by the patient. Adherence to the training programmes will be assessed through a RehaCom therapist account for the cognitive training and through a Polar Flow for Coach account for the motor training.

### Primary outcome parameter

The primary outcome measure is the digit span backwards test [33], which assesses working memory. The examiner produces a list of digits at a rate of one digit per second and patients have to repeat the list in reverse order. Two trials are presented for each span length. If patients fail two trials of the same span length, the test is ended. The test starts with a list of two items and gradually increases to a maximum of eight items. Based on clinical experience, we will consider an improvement of one point on this test a clinically significant difference. This test will be carried out at the start of the trial, after 12 weeks of training, at 24 weeks and 64 weeks.

### Secondary outcome parameters

The following secondary clinical outcome measures will be assessed: Expanded Disability Status Scale (EDSS), Brief International Cognitive Assessment for Multiple Sclerosis [45] (BICAMS), Backward Corsi [34], 6-Minute Walk Test [46] (6MWT), 25-Foot Walk Test (25FWT), 9-Hole Peg Test (9HPT), Hospital Anxiety and Depression Scale [47] (HADS), Fatigue Scale for Motor and Cognitive functions [48] (FSMC), Visual Analogue Scale [49] (VAS) on the impact of perceived cognitive symptoms on daily life, Cognitive & Leisure Activity Scale [50] (CLAS) and Godin Leisure-Time Exercise Questionnaire [39] (GLTEQ). Furthermore, the 36-Item Short Form Survey [51] (SF-36) and Multiple Sclerosis Impact Scale-29 [52] (MSIS-29) will be completed by Dutch-speaking participants and the Sclérose En Plaques-59 [53] (SEP-59, a combination of the SF-36 and MS-specific questions) will be completed by French-speaking participants. All tests will be carried out at the start of the trial, after 12 weeks of training, at 24 weeks and 64 weeks.

Furthermore, MRI and EEG data will be collected at the start of the trial and after 12 weeks of training, with an additional MRI exam at 24 weeks.

### MRI of the brain

MRI analyses will be carried out at the Universitair Ziekenhuis Brussel on a GE SIGNA Premier 3T MRI scanner using a 48-channel head coil. Scans will be performed at the start of the trial, after 12 weeks of training and at 24 weeks. The following images will be acquired: a high-resolution 3D T1 scan (echo time (TE) 3.0ms; repetition time (TR) 7.3ms; flip angle = 12°; field of view = 256 × 256mm^2^; acquisition matrix = 256 × 256; 180 slices; slice thickness = 1.0mm; bandwidth = 244.1 Hz/pixel; acceleration factor 1.5 × 1.5; scanning time = 4m08s. Images will be reconstructed to a 512 × 512 × 360 matrix), a high-resolution 3D T2 FLAIR scan (TR = 7800ms; TE = 124ms; echo train length (ETL) = 222; field of view = 256 × 230mm^2^; acquisition matrix = 256 × 256; 170 slices; slice thickness = 1.0mm; bandwidth = 390.6 Hz/pixel, acceleration factor 4.6 using HyperSense; scanning time = 4m13s. Images will be reconstructed to a 512 × 512 × 172matrix), a multi-shell high angular resolution diffusion-weighted imaging dataset (HARDI, TR = 4716s; TE = 71.4ms; field of view = 220 × 220mm; acquisition and reconstruction matrix = 128 × 128; 80 slices; slice thickness/gap = 1.7/0.0mm; 16 directions with *b*=700 s/mm^2^, 30 directions with *b*=1200s/mm, 50 directions with *b*=2800s/mm^2^; 5 *b*=0 images interleaved, hyperband factor =3, bandwidth = 3906.25 Hz/pixel; scanning time = 7m52s) along with a phase reversed volume with *b* = 0 and a 3D-QALAS (TR = 5000ms; TE = 1.9ms; inversion time = 100ms; ETL = 150; flip angle = 4; field of view = 256 × 256 × 150mm; cycle time = 900ms; bandwidth = 454.5Hz/pixel; acquisition and reconstruction resolution = 1.0 × 1.0 × 1.0mm; acceleration factor 2.6 using HyperSense; scanning time = 7m38s), to acquire quantitative maps and perform synthetic MRI. The diffusion directions will be calculated using a bipolar electrostatic repulsion model, as implemented in mrtrix [54–56]. Fibre cross-section (FC) and apparent fibre density (FD) will be investigated in white matter through a fixel-based hARDI analysis [57]. The following parameters will be assessed: diffusion tensor image (DTI) parameters (fractional anisotropy (FA) and mean diffusivity (MD)) derived from the *b*=700 s/mm^2^ images, graph theoretical metrics (average path length, edge strength, clustering coefficient, smallworldness and modularity, ratio of inter- versus intrahemispheric connectivity strength), lesion volume, cortical volume and volumes of white matter and deep grey matter. See below for more details.

### EEG

EEG recordings will be made at the start of the trial and after 12 weeks of training. Measurements will be obtained using a portable recording setup with 64 EEG sensors, with horizontal and vertical eye movements and electrocardiography (ECG) leads. Sampling frequency will be 512 Hz. Resting-state EEGs will be recorded, as well as task-related EEGs with the following paradigms: auditory P300 paradigm [58], n-back test [59] and adjusted symbol digit modalities test (SDMT) [60]. Event-related potentials, as well as the following graph theoretical metrics will be assessed: average path length, edge strength, clustering coefficient, smallworldness and modularity and ratio of inter- versus intrahemispheric connectivity strength. See below for more details.

### Sample size

We calculated the minimal sample size with G*Power [61], using an ANOVA design (repeated measures, within-between interaction) with a power of 95% and an alpha error rate of 5%. Under the assumption of a large effect size (*f* = 0.40, based on the change in digit span backwards score after cognitive training [62]) and moderate correlation between the pre- and post-training measurements (*r* = 0.50), the required sample size is 30. Under the less favourable assumption of a medium effect size (*f* = 0.25) and a small correlation among repeated measurements (*r* = 0.30), the required total sample size is 90. In this study, we chose a total sample size of 90, with 30 subjects in each group.

### Blinding and statistical methods

The study will be blinded: patients will not be made aware of the rationale and predictions of the study (pseudo-blinding). The randomisation procedure and the teleconsultations will be carried out and communicated to participants by a trained MS nurse, while the baseline and follow-up testing and analyses are carried out by an investigator who is blinded to the treatment allocation of the patients. Unblinding will only be done in the unlikely case of serious adverse events.

Differences between the different treatment groups in the clinical endpoints compared to baseline will be assessed with a one-sided ANOVA repeated measures test with a within-between interaction. A type I error probability of 0.05 will be used for the main effects and interaction effects. We will correct for multiple comparisons in the post hoc analyses via a family-wise error rate. Per-protocol analyses will be performed, and to correct for missing data, analyses will be repeated using the last observation carried forward method. In case of dropout before the second follow-up testing at 24 weeks, the differences in clinical endpoints at week 12 compared to baseline will still be assessed.

Furthermore, we will identify mechanisms of improvement using graph theoretical analysis. We will pre-process the EEG data in the Oxford Centre for Human Brain Activity (OHBA) software library (OSL 2.0), first using opt (OSL’s pre-processing pipeline), then using oat (OSL’s analysis tool). We will use independent component analysis to denoise recordings. Then, we will identify and regress out artefactual independent components by their degree of correlation with electrooculography (EOG) and ECG and by their degree of kurtosis (eliminating both extreme high and low kurtosis). Then, we will calculate a panel of potential EEG markers of cognitive performance, derived from network EEG analysis. From the pre-processed EEG, we will extract 4-second epochs. This epoch length is an adequate compromise between frequency resolution and minimisation of data loss. These epochs will enter a window-based network construction with the electrodes treated as nodes, calculated using the mutual information edge detection. From the diffusion-weighted imaging dataset, we will derive structural network information via probabilistic tractography and a pre-defined atlas. Graph theoretical metrics calculated for both functional (EEG) and structural (diffusion weigthed imaging) measures allow us to summarise the functional and structural networks in a small number of features. Next to traditional features (average path length, edge strength, clustering coefficient, smallworldness and modularity), ratio of inter- versus intrahemispheric connectivity strength will be assessed.

Finally, we will identify predictors of treatment response by using logistic regression. To account for possible non-linear correlations, we will use machine learning, more specifically random forest classifiers.

## Discussion

The treatment of cognitive impairment in MS is an important unmet need. While cognitive rehabilitation has been proven to be effective in improving cognitive performance in MS [7–9], studies with larger sample sizes, inclusion of patients with progressive disease, assessment of the effects on daily life and long-term follow-up data are still lacking [6]. Furthermore, research in the elderly indicates that combined cognitive-motor rehabilitation may be more effective than single cognitive or motor training [23]. We set up a randomised controlled clinical trial to assess whether a combined cognitive-motor telerehabilitation programme improves working memory compared to only cognitive and only motor telerehabilitation in PwMS.

Some aspects of the trial need clarification. First, we have chosen not to include a passive control group, since the effectiveness of cognitive rehabilitation in multiple sclerosis has already been proven [7–9], and the goal of this study is to evaluate whether a combined cognitive-motor training is more effective than single cognitive or motor training in improving cognitive performance. Second, our primary outcome measure is the digit span backwards, a measure of working memory, since the cognitive part of our training programme is focused on working memory improvement and studies have shown that improvement of cognitive performance seems to be limited to the functions that were trained [23]. Impairments in working memory occur frequently in MS, affecting patients in both the earliest and later stages of the disease [63]. Third, while letting the patient choose between different aerobic activities increases variability of the motor training, it allows for an increased feasibility of the study and motivation of the patient, as well as increased clinical applicability. Finally, despite the known limitations of age-predicted maximum heart rate equations [40], we have chosen not to carry out a maximal exercise test to calculate the heart rate zone, since the planned testing and training are already very elaborate and taxing for the patient and the goal of the heart rate monitoring is to support the patient in preventing that the training intensity is too high or too low, rather than to construct a strict training regimen of a specific intensity. Since MS can be associated with an attenuated heart rate response to exercise due to autonomic dysfunction, patients will also be using a Rating of Perceived Exertion scale (RPE [44]) to assess training intensity.

Research on combined cognitive-motor training in multiple sclerosis is still lacking. Our trial is unique since it is the first to compare the effect of combined cognitive-motor training to both single cognitive and single motor training on cognition in MS. Furthermore, the training is completely home-based, thereby eliminating transportation and reducing therapist time, while enabling the patient to carry out the training independently at any time of the day. These advantages increase the potential applicability of this intervention in daily practice. Finally, our study will include a large sample of PwMS, with both a relapsing-remitting and progressive disease course, and collection of data on long-term follow-up, mechanisms of improvement and predictors of treatment response.

### Trial status

Recruitment will start in the summer of 2022 and will be completed in the summer of 2025.

Protocol version 2.2, dated 18 July 2022.

### World Health Organization Trial Registration Data Set

**Table:** 

Data category	Information
Primary registry and trial identifying number	clinicaltrials.gov, NCT05355389
Date of registration in primary registry	2 May 2022
Secondary identifying numbers	Fonds Wetenschappelijk Onderzoek (FWO), 1SD5322N
Source(s) of monetary or material support	Fonds Wetenschappelijk Onderzoek (FWO)
Primary sponsor	Universitair Ziekenhuis Brussel/Vrije Universiteit Brussel Laarbeeklaan 101 - 1090 Jette, 02 477 41 11 Pleinlaan 2 - 1050 Brussel
Secondary sponsor(s)	/
Contact for public queries	Delphine Van Laethem, delphine.van.laethem@vub.be Guy Nagels, guy.nagels@uzbrussel.be
Contact for scientific queries	Delphine Van Laethem, delphine.van.laethem@vub.be Guy Nagels, guy.nagels@uzbrussel.be
Public title	Cognitive-motor telerehabilitation in multiple sclerosis
Scientific title	Cognitive-motor telerehabilitation in multiple sclerosis
Countries of recruitment	Belgium
Health condition(s) or problem(s) studied	Multiple sclerosis
Intervention(s)	Cognitive training: working memory training with RehaCom Motor training: patient-tailored aerobic training *Experimental*: combination of cognitive and motor training *Active comparators*: single cognitive and single motor training
Key inclusion and exclusion criteria	*Ages eligible for study*: 18-65 years *Sexes eligible for study*: both *Accepts healthy volunteers*: no *Inclusion Criteria*: clinically definite multiple sclerosis (revised McDonald criteria 2017), Expanded Disability Status Scale (EDSS) below 6.0, digit span backwards z-score between [-3 and -0.5] standard deviations below the median of the normative values, age between 18 and 65, able to safely perform motor rehabilitation in the home situation (assessed by rehabilitation physician and/or occupational or physiotherapist) *Exclusion Criteria*: cognitive rehabilitation within six months before inclusion, inpatient multidisciplinary rehabilitation program within three months before inclusion or planned inpatient program during trial, start of or switch in immunomodulator treatment within three months before inclusion, less than one month post-exacerbation, major psychiatric or medical disorder that could influence cognitive functions, combined vision with optimal correction below 0.6 on Snellen Visual Acuity Test, unable or unwilling to undergo EEG or MRI
Study type	Pseudoblinded* parallel randomised controlled intervention trial *subjects are blinded to study rationale, investigators are blinded to treatment allocation
Date of first enrolment	1 September 2022
Target sample size	90
Recruitment status	Recruiting
Primary outcome(s)	Change in digit span backwards post- compared to pre-treatment
Key secondary outcomes	Change in other cognitive scores (BICAMS, backward Corsi), physical scores (EDSS, 6MWT, 25FWT, 9HPT), questionnaires (HADS, FSMC, VAS, SF-36, MSIS-29, SEP-59, GLTEQ, CLAS), MRI and EEG parameters

## Supplementary Information

**Additional file 1.** SPIRIT Checklist.

## Data Availability

Only researchers that are directly involved in the project will have access to the final, pseudonymised dataset. Researchers interested in a collaboration are welcome to contact the senior authors.

## Abbreviations

6MWT: 6-Min Walk Test
BICAMS: Brief International Cognitive Assessment for Multiple Sclerosis
CI: Cognitive impairment
CLAS: Cognitive and Leisure Activity Scale
DTI: Diffusion tensor image
EDSS: Expanded Disability Status Scale
ECG: Electrocardiography
EEG: Electroencephalography
EOG: Electrooculography
ETL: Echo train length
FA: Fractional anisotropy
FSMC: Fatigue Scale of Motor and Cognitive functions
GLTEQ: Godin Leisure-Time Exercise Questionnaire
25FWT: 25-Foot Walk Test
9HPT: 9-Hole Peg Test
HADS: Hospital Anxiety and Depression Scale
MD: Mean diffusivity
MRI: Magnetic resonance imaging
MS: Multiple sclerosis
MSIS-29: Multiple Sclerosis Impact Scale-29
OHBA: Oxford Centre of Human Brain Activity
PwMS: Persons with multiple sclerosis
RPE: Rating of Perceived Exertion
SDMT: Symbol Digit Modalities Test
SEP-59: Sclérose En Plaques-59
SF-36: Short Form-36
TE: Echo time
TR: Repetition time
VAS: Visual Analogue Scale
